# Comparative Study of Chemically Treated Sugarcane and Kevlar Fiber to Develop Brake Resistance Composites

**DOI:** 10.3390/molecules28124861

**Published:** 2023-06-20

**Authors:** Vikas Mehta, Naresh Kumar, Ali Algahtani, Vineet Tirth, Tawfiq Al-Mughanam, Kwok-Wing Chau

**Affiliations:** 1 Department of Civil Engineering, Keimyung University, 1095 Dalgubeol-daero, Dalseo-gu, Daegu 42601, Republic of Korea; 2 Department of Mechanical Engineering, Green Hill Engineering College, Solan 173229, HP, India; 3 Department of Mechanical Engineering, King Khalid University, Abha 61421, Saudi Arabia; 4 Research Center for Advanced Materials Science (RCAMS), King Khalid University, Guraiger, Abha 61413, Saudi Arabia; 5 Department of Mechanical Engineering, College of Engineering, King Faisal University, P.O. Box 380, Al-Ahsa 31982, Saudi Arabia; 6 Department of Civil and Environmental Engineering, The Hong Kong Polytechnic University, Kowloon, Hong Kong, China; dr.kwok-wing.chau@connect.polyu.hk

**Keywords:** friction materials, natural fiber, non-asbestos textiles, sugarcane fiber, thermal stability

## Abstract

Recently, much research has revealed the increasing importance of natural fiber in modern applications. Natural fibers are used in many vital sectors like medicine, aerospace and agriculture. The cause of increasing the application of natural fiber in different fields is its eco-friendly behavior and excellent mechanical properties. The study’s primary goal is to increase the usage of environmentally friendly materials. The existing materials used in brake pads are detrimental to humans and the environment. Natural fiber composites have recently been studied and effectively employed in brake pads. However, there has yet to be a comparison investigation of natural fiber and Kevlar-based brake pad composites. Sugarcane, a natural fabric, is employed in the present study to substitute trendy materials like Kevlar and asbestos. The brake pads have been developed with 5–20 wt.% SCF and 5–10 wt.% Kevlar fiber (KF) to make the comparative study. SCF compounds at 5 wt.% outperformed the entire NF composite in coefficient of friction (µ), (%) fade and wear. However, the values of mechanical properties were found to be almost identical. Although it has been observed that, with an increase in the proportion of SCF, the performance also increased in terms of recovery. The thermal stability and wear rate are maximum for 20 wt.% SCF and 10 wt.% KF composites. The comparative study indicated that the Kevlar-based brake pad specimens provide superior outcomes compared to the SCF composite for fade (%), wear performance and coefficient of friction (Δμ). Finally, the worn composite surfaces were examined using a scanning electron microscopy technique to investigate probable wear mechanisms and to comprehend the nature of the generated contact patches/plateaus, which is critical for determining the tribological behavior of the composites.

## 1. Introduction

The demand for brake friction materials is rising significantly in the modern day because of a rapid increase in the number of fast automobiles. To accomplish this objective, however, an effective braking system is necessary. Brake friction materials play a key role in achieving the competing performance criteria of friction and wear throughout a wide range of operating conditions. The current materials used in brake pads harm humans and the environment. Trending materials like Kevlar and asbestos are a cause of cancer. As a result, a brake friction composite comprises different materials, such as fibers, fillers, binders and friction modifiers. After asbestos was removed from brake friction materials due to its carcinogenic impact, the evolution of non-asbestos organic friction compounds was halted. Non-asbestos brake friction materials, on the other hand, have been technologically developed. Despite this, they have been prone to several braking-related performance peculiarities that needed to be more practically non-existent in asbestos-based brake friction composites. Attempts are being undertaken to improve the frictional performance of brake friction materials in various ways, principally by including novel chemicals or their combinations into the composition. Different waste materials, natural fibers and nanomaterials have been asserted to have the potential to be employed in automobile brake friction composites in this regard. Today’s investigation remains to progress in engineering practice to discover the alternative to Kevlar and asbestos. According to WHO reports, these substances harm humans and the environment [1]. These substances are one reason for cancer and have taken millions of lives [1,2,3]. As a substitute for asbestos, several scholars have proposed various alternatives, such as aluminum, steel, wollastonite, Lapinus and carbon [4,5,6,7]. However, Kevlar is still a brake friction substance without superior options [8,9]. The trending friction substance displays the same effect as asbestos. Furthermore, it has limitations, such as inadequate reuse, more cost, adversity to humans and the environmental condition and non-recyclability [10,11,12]. Natural fiber (NF) has become trendy because of its environment-friendly nature, superior mechanical properties, lightweight nature and accessibility [13,14,15,16,17,18]. Several articles have demonstrated the potential of NF polymer composites [19,20,21,22,23,24]. However, a few shortcomings of NF can be overcome by chemical treatment [25,26,27,28,29]. SCF is the utmost popular crop worldwide [30]. SCF can be used as polymer composite reinforcement due to its abundant availability, low density, low pre-treatment costs and good mechanical properties. SCF is an agricultural waste that is composed after the extraction of liquor. Moreover, to being nearly boundless, it is generally wasted in countries that cultivate it, which distinguishes a fiber of meager cost. It is usually used in ethanol, building boards and polypropylene polymer composites. It is made up of three major components: rind (22%), pith (5%) and fiber (73%). Mechanical parameters, such as tensile strength, Young’s modulus and density, range from 20 to 50 MPa up to 2.7 GPa and 1.28 g/cc, respectively [31,32,33]. The ingredients of SCF are lignin to 18–24%, hemicelluloses 20–25%, cellulose 45–55% and ash 1.18%. It had a vital role in the life of humans during ancient times. Therefore, SCF considers brake pad friction material because of its excellent mechanical properties. The SCF provides good thermal productivity, which can be applied to create brake pad composites. The role of such property by SCF gives a unique brake system performing in the motorized purpose if suitably optimized. Hence due to its excellent mechanical properties, an attempt to get the brake pad with different percentages of 5–20 wt.% SCF and 5–10 wt.% Kevlar fiber (KF) by weight in the total matrix mixture for optimization. The tensile strength, flexural strength, density, hardness test, impact test, wear, coefficient of friction, absorption and optimized composite percentage are well suited for making brake pad material. Several investigators have studied natural fibers frictional laminate materials in recent years and have proposed that the multiple benefits of natural fibers will give a less affordable and much more environmentally friendly substitute to expensive fibers, such as Kevlar, utilized in the brake friction material industry. Maleque et al. [16] discovered that adding 5% coir fibers to frictional compositions led to maximum wear resistance. The tribological characteristics of flax fiber-reinforced friction composites were investigated by Z. Fu et al. [27]. They discovered that 5.6 vol.% flax fibers stabilize the friction coefficient and increase wear resistance at high temperatures. Y. Liu et al. [28] investigated the effect of abaca fiber on phenol resin-based materials’ wear and friction characteristics. They found that the number of fibers and their length may significantly improve tribological properties. Furthermore, sisal fiber [29], hemp fiber [34], bamboo fiber [35], kenaf fiber [36] and, most recently, cow dung fibers [37] have been shown to increase tribological properties. Tej Singh et al. [38] study comparing pineapple fiber, and Kevlar offers good physical tribological properties. However, the comparison is made with constrained criteria. Natural fibers can be used to replace asbestos and other harmful materials. Sugarcane fiber, generated from the sugarcane plant and causing environmental degradation due to direct dumping on open areas, is one of the most abundant raw resources. Although sugarcane fiber-reinforced composites have been found to have acceptable physical and mechanical characteristics, the study refers to research on using these fibers in the development of limited brake friction materials [30,31,32,33]. The brake friction material is a cost-effective and environmentally friendly alternative to synthetic fibers, such as Kevlar, in the automobile sector. Natural sugarcane fibers were used as phenol-formaldehyde resin reinforcement to create the hybrid composite. Sugarcane fiber-based friction composites were also tested against Kevlar fiber-reinforced composites. To conduct the contrast investigation, the brake pads were made using 5–20 wt.% SCF and 5–10 wt.% Kevlar fiber (KF). The findings of the studies show that the new composite has adequate friction and wear performance, making it a good choice for brake components. A comparative investigation discovered that Kevlar-based brake pad specimens performed slightly better in terms of % fade, coefficient of friction and wear performance than Sugarcane composites. Mechanical property values were determined to be almost equal. The wear rate and thermal stability were maximum for composites containing 20% sugarcane fiber and 10% Kevlar.

## 2. Results and Discussion

### 2.1. Physical, Chemical, Mechanical and Tri-Biological Characterization of SCF/KF Compounds

Physical, mechanical and chemical properties achieved for the duration of the experiment are presented in Table 1. During the test, it was examined that an increased proportion of SCF porosity also increased in the matrix. However, the range of SCF composites’ porosity lies between 3.85% and 4.4%, while for Kevlar composites, it varies between 3.80% and 3.90%. It occurs because of the insufficient blending of the ingredients in the polymer matrix. However, with the increase of fibers, water absorption also increased. Although the range of water absorption of advanced composites lies between 2.70% and 3.27%, for SCF and Kevlar composites, it lies between 2.45% and 2.60%. The porosity and water absorption trend can be correlated. At the lowest porosities (SCF = 3.80% and KF = 3.85%), the water absorption value is also the lowest (SCF = 2.70% and KF = 2.45%). At the highest value of porosities (4.4% and 3.90%), the water absorption has been noted highest (SCF fiber = 3.27% and Kevlar = 2.60%). However, compressibility is expected at the lowest porosity value (SCF composite = 1.48% and Kevlar composite = 1.45%).

The greater porosity demonstrates maximum compressibility for developed composites [39,40,41]. In the polymer composite, the density decreases (SCF = 2.80–2.70 g/cm^3^ and Kevlar = 2.78–2.50 g/cm^3^) as SCF and Kevlar ratio increases. It may arise due to the lightweight (CF and Kevlar) replacing the heavyweight material (barium sulfate). The mechanical properties decrease with the rise of SCF and Kevlar because lightweight materials are replaced by heavy materials (barium sulfate). Therefore, as the percentage of fiber increases, the mechanical properties decrease simultaneously. However, a discrepancy in amounts of acetone extraction has been noted highly a smaller amount (SCF = 0.62–0.75% and Kevlar = 0.60–0.65), which exhibits the appropriate curing of the established friction items. The ash substances were reduced (SCF = 83.87–78.74% and Kevlar = 85.80–84.85%) with a rise in wt. (%) of SCF and Kevlar. However, the five wt.% displays the maximum amount of ash content. The hardness increases with the increased SCF and KF in the developed samples. The cause for reducing ash substances and a rise of SCF and KF is lower resistance fiber has been substituted high resistance barium sulfate. The hardness decreases with an increasing proportion of SCF and KF in composites (SCF 115.9–113.7 and Kevlar 113.6–109.3). However, the tensile strength has decreased (SCF 17.50–12.98 MPa and Kevlar 17.98–16.70 MPa). The maximum tensile strength value has been found at 5 wt.% composites (FS-1 = 17.50 MPa and KV-1 = 17.98 MPa) as shown in Table 1. Proof stress has been noticed highest for FS-4 (5 MPa) and KV-2 (4.7 MPa). In comparison, flexural strength demonstrates its uppermost value for FS-1 and KV-1 (SCF = 64.37 MPa and Kevlar = 68.29 MPa), and flexural Modulus also presents its highest value at FS-4 and KV-1 (SCF = 2.80 GPa and Kevlar = 2.45 GPa) [41] although an increase in SCF proportion in the composite heat swelling increases. The cause might be the extremely high temperatures. NF attempts to spread out and generate a few warm gases in the compounds. FS-1 (1.65) and KV-1 (1.5) present the minor extension in Table 2. The tensile modulus represents the maximum value for FS-1 (4675 MPa) and KV-1 (4832 MPa), including the lowest FS-4 (4174 MPa) and KV-2 (4756 MPa). It specifies that an improving percentage of SCF leads to a non-uniform matrix. The impact energy shows a better value for FS-1 (0.464) and KV-1 (0.470) and the lowest possible for KV-2 (0.465) and FS-4 (0.385).

**Table 1:** Descriptions of SCF and KV compounds.

Properties	Standard Applied [40,41,42,43,44]	KV-1	FS-1	KV-2	FS-2	FS-3	FS-4
% Porosity	JISD 4418 standard	3.8	3.85	3.9	3.95	3.43	4.4
% Ash content	ASTM D570-98	85.8	83.87	84.85	82.6	79.63	78.74
% Water absorption	ISO 6310 standard	2.45	2.7	2.6	2.75	3.2	3.27
% Acetone extraction	ASTM D494 standard	0.6	0.62	0.65	0.67	0.7	0.75
Density (g/cm^3^)	ASTM C271/C271 M-16	2.8	2.75	2.7	2.7	2.65	2.62
% Compressibility	ISO 6310	1.45	1.48	1.53	1.55	1.6	1.65
Hardness (HRR)	Rockwell-R scale	115.9	113.6	113.7	111.4	110.8	109.3
% Heat swelling	SAE J 160 JNU80	1.5	1.65	1.75	1.95	2.05	2.1
Impact energy (J)	ASTM D256	0.47	0.465	0.464	0.45	0.39	0.385
Shear strength (kgf)	ASTM D732	1560	1423	1510	1490	1367	1320
Tensile strength (MPa)	ASTM E8	17.98	17.5	16.7	16.2	13.39	12.98
Flexural strength (MPa)	ASTM D790	68.29	64.37	66.76	61.65	58.34	57.28
Tensile modulus (MPa)	ASTM E8	4832	4675	4640	4543	4376	4174
Flexural modulus (GPa)	ASTM D790	2.59	2.4	2.45	2.65	2.7	2.8
Failure strain (%)	ASTM E8	1.65	1.7	1.6	1.85	2.05	2.1
Proof stress (MPa)	ASTM E8	4.5	4.75	4.7	4.8	4.98	5
Ultimate compressive strength (MPa)	ASTM E8	178.7	172.5	180.6	164.3	160.7	158.3

The compressive strength has been observed to be highest for FS-1 (180.6 MPa) and KV-1 (178.7 MPa) and lowest for FS-4 (158.3 MPa) and KV-2 (172.5 MPa) [42]. The shear strength has been observed maximum for FS-1 (1510 MPa) and KV-1 (1560 MPa); it can be conceivable that a rise proportion of 5–10% of SCF could act as a binder although 15–20% fiber compound has been presented, non-uniform medium revealed its lowermost amount for FS-1 and KV-1 and uppermost for FS-4 and KV-2 [43,44]. The flexural strength, failure strain and flexural modulus have demonstrated their maximum values at FS-1.

### 2.2. Tri-Biological Properties of Samples

In the tri-biological test, the favorable values of the coefficient of friction are shown in Table 2.

### 2.3. Effect of Friction (µ) in Fade and Recovery Phases

**Figure 1:**
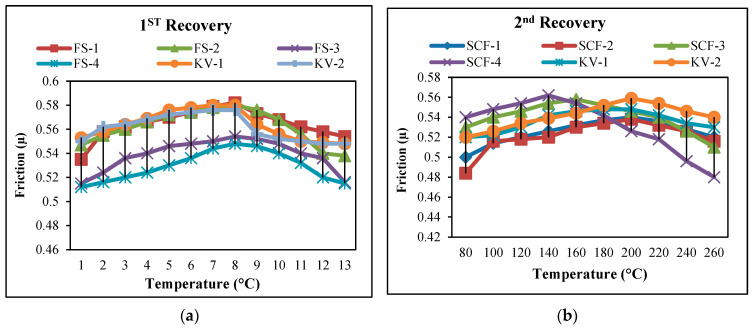
(**a**) 1st Recovery cycles of developed samples; (**b**) 2nd Recovery cycles of developed samples.

**Figure 2:**
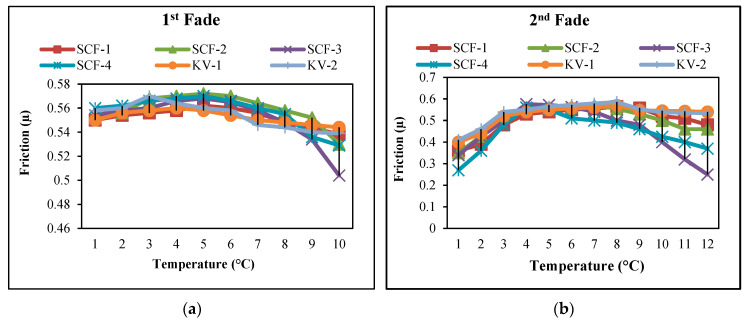
(**a**) 1st Fade cycles of developed samples; (**b**) 2nd Fade cycles of developed samples.

The coefficient of friction is increased to 175 °C for all SCF composites and Kevlar-based composites. It has been observed that, once the temperature reaches 175 °C, the SCF-3 and SCF-4 compounds reduce abruptly even though the rate of SCF-1 and SCF-2 rises to the temperature of 200 °C and then decreases very slowly. However, it has been noticed that KV-1 and KV-2 fiber compounds have minimal alterations, demonstrating outstanding sustainability, as illustrated in Figure 1 and Figure 2. In the secondary fade cycle for SCF-1 and SCF-2 compounds, it has been noticed that the coefficient of friction rises temp. 250 °C, while for SCF-3 and SCF-4, it rises to 195 °C and later starts to decline (steeply), similarly to 1st fade cycle. For KV-1 and KV-2, the coefficient of friction is identical. In the recovery cycle, µ has been improved up to 220 °C for all samples afterward; µ leads to a steady decline for SCF-1 and SCF-2; however, SCF-3 and SCF-4 decreased rapidly. For secondary recovery, µ for SCF-1 and SCF-2 raised to 200 °C; later, it appeared to be a gradual decline, and for SCF-3 and SCF-4, the µ started to fall rapidly after 150 °C temp.

### 2.4. Frictional Stability (μ_S_) and Variability (μ_V_) Coefficient Performance

**Figure 3:**
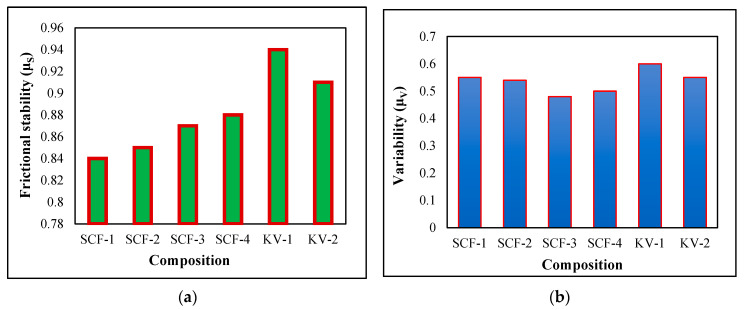
(**a**) Frictional behavior of μ_S_ (stability coefficient); (**b**) μ_V_ (Variability coefficient) of developed composites.

The μ_S_ and μ_V_ coefficients have been calculated through Equations (1) and (2) [44,45,46]. During the test, it was determined that the stability value reduces with a raised proportion of SCF, while the importance of variability rises with a high SCF ratio. The limit for peace has been noted between 0.88 and 0.84, and for variability, the range has been reported between 0.49 and 0.56, as presented in Figure 3a,b.

Stability = performance μ/max μ(1)

Variability = variation in (Δ) μ/performance μ(2)

SCF-1 composite demonstrates its maximum value of 0.56 and lowest value of 0.49 for variability. The Kevlar composite’s variability and stability rates lie between 0.57 and 0.59 and 0.91 and 0.94, respectively. The lower value of μ_v_ and higher value of μ_S_ is considered adequate for whole frictional compounds. It has been observed that the value of %F rises with an increasing proportion of SCF and KF Composite.

### 2.5. Fade (F) (%) and Recovery (R) Performance Analysis

The calculations formula for finding R and F (%) are:

%F = performing μ − fade μ/performing μ × 100(3)

%R = recovery μ/performing μ × 100(4)

**Figure 4:**
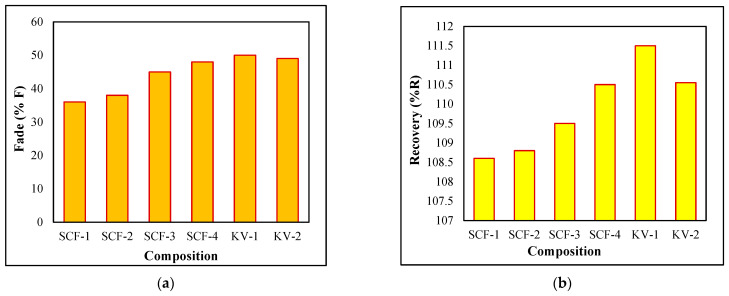
Frictional Behavior of %Fade (**a**) and %Recovery (**b**) of developed composites.

It has been observed that the fade value varies between 37.2 and 47.6 for SCF composites, and Kevlar composites vary from 54.6 to 54.7, as indicated in Figure 4. However, recovery rises with the increase in SCF proportion and lies in 108.6–110.6%. SCF-4 reveals the ultimate value, but SCF-1 shows the most negligible value of %R. The lowest fade values are observed, i.e., 37.2 for SCF-1, and the highest recovery, i.e., 110.6 for SCF-4, as shown in Figure 4. The %R of KF based also increased with the proportion of Kevlar (KV-1 = 110.8, KV-2 = 111.3). The most negligible value of %F and the utmost importance of %R is considered favorable for the entire friction experiments. The SCF-1 and SCF-2 lower proportion-based SCF compounds present the most negligible fade value (%F), i.e., 28% lower than SCF-3 and SCF-4. The enhancement in fade (%F) with reduction has been noticed in NF substances.

The rising proportion of SCF in polymer mixtures creates a discrepancy matrix; in tribal experiments, it may be caused to generate extra wear particles and debris (act as the third body) among the frictional boundaries and enhance the performance [46]. However, the improved fiber makes an inhomogeneous matrix that produces debris and performs as wear particles throughout the friction test to improve friction [34,39,40,46]. Although, with increasing the proportion of SCF and KF, the values of fade and recovery were observed to be enhanced. The accumulation of SCF and KF in polymer compounds indicates easier shear and degradation. It improves the values of %F and %R, as described in the literature [46,47,48,49,50].

### 2.6. μ_P_, μ_R_, μ_F_ and Δ_μ_ Performance of SCF Compounds

**Figure 5:**
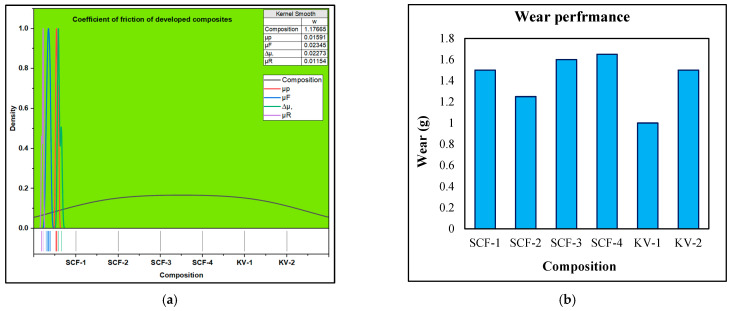
(**a**) COF performance of μ of the developed compound; (**b**) wear performance of μ of the developed compound.

The performance of the coefficient of friction has been presented in Figure 5. Several parameters like μ_p_, μ_F_, μ_R_ and Δ_μ_ have been investigated during the test. It has been observed that the performance of μ and F coefficient decreased with the increasing proportion of SCF in polymer compounds. However, the ranges lie between 0.360 and 0.284 for μ_F_ and 0.549 and 0.523 for μ_P_ at more than 200 °C. The friction values reduce as the NF compounds and phenolic resin decompose [34,39,40,46,47,48]. The μ_R_ value increased (0.586–0.590) in SCF proportion in developed mixtures and KF composite (0.655–0.641)—the value of Δ_μ_ increases as the SCF proportion rises in the developed brake pad models. The Δ_μ_ value for SCF-1 and SCF-2 fibers grew compound limits between 0.220 and 0.223, whereas SCF-3 and SCF-4 samples were in the 0.228–0.235. However, due to the method of tribo-produced boundaries (reorganization and fragmentation), the Δ_μ_ value could be enhanced, leading to the creation of friction film. Secondly, raising the proportion of pure fibers in compounds reduced the adhesion strength with phenolic resin. Therefore, it forms debris and discontinuity information and performs as a third party, enhancing friction values [34,39,40]. The μ_F_ and μ_p_ for KV-1 values were noticed as 0.575 and 0.395, respectively, although KV-2 has shown fall values, i.e., 0.570 and 0.392. The decreasing tendency in the values may be caused by tribo-interfaces process [51]. The performance of µR for KV-1 and KV-2 is 0.655 and 0.641, respectively. The Δ_µ_ value has been noticed at 0.186 and 0.197, respectively, for KV-1 and KV-2. However, SCF-1 shows 60% less fluctuation in friction (Δ_µ_) than SCF-4 compounds. However, the value of SCF-1 demonstrates a 5% better value than SCF-4 for μp, indicating that with an increasing proportion of SCF, good fiber values begin to reduce. The μ_F_ value for SCF-1 and SCF-2 compounds must be 27% greater than that of SCF-3 and SCF-4; however, for μ_R_ (maximum discount is beneficial), the value rises with a rising proportion of SCF. The SCF-1 and SCF-2 show enhanced values. It has also been seen in the KF case that KV-1 indicated improved outcomes for μ_p_, μ_F_, Δ_µ_ and µ_R_ than that of KV-2.

### 2.7. Wear Performance

**Figure 6:**
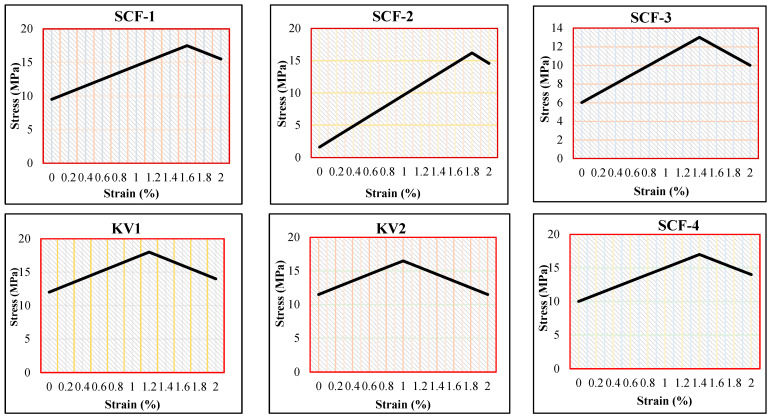
Tensile stress-strain rate of developed compound.

The wear test has observed the wear rate increase with an increasing proportion of SCF and Kevlar in composites although the wear range was found between 1.1 and 1.7 g for SCF and 1.0–1.08 for Kevlar-based polymer composites, as shown in Figure 6. SCF-1 composite has established the minimum wear value, whereas SCF-4 has demonstrated the maximum wear value. KV-1 has shown better results than KV-2. The increased percentage of sugarcane fiber does not mix properly with other ingredients in a polymer composite, which leads to an increase in the wear rate [34,39,40]. The velocity of wear performance was 4.2 m/s with a sliding distance of 1200 m. The tensile stress-strain rate of the developed composite has been presented in Figure 6.

### 2.8. Worn Surface Assessment

**Figure 7:**
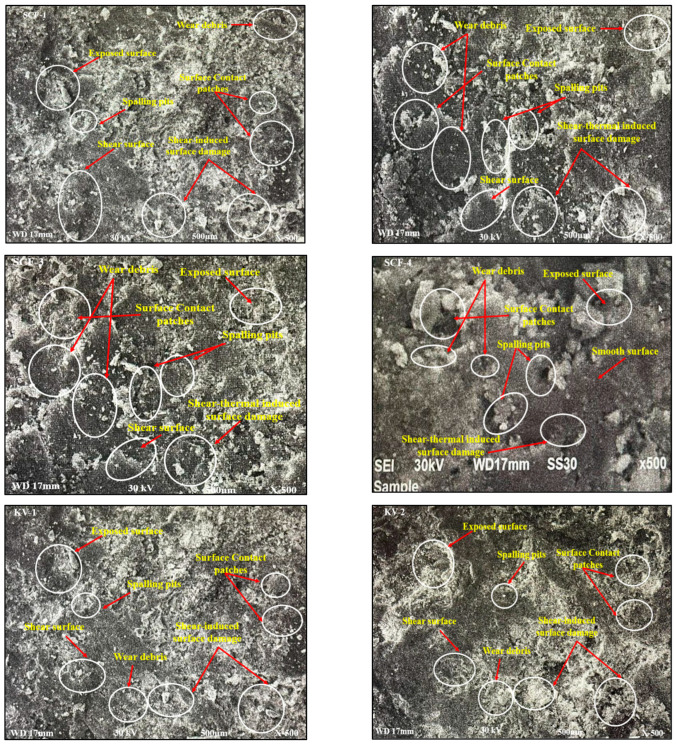
SCF-1, SCF-2, SCF-3, SCF-4, KV-1 and KV-2 composites with worn-out surface morphology.

According to the worn surface research, the formation of primary and secondary contact patches/plateaus is one of the most crucial factors in determining the tribological performance of the composite, along with abrasive and adhesive wear processes. From scanning electron microscope (SEM), it has been observed that small pits, contact plateaus, small debris and more smooth areas created on the exterior portion of SCF-1 and SCF-2 compounds where more pits, fewer interaction plateaus, high derbies and high rough surfaces arise in the external part of SCF-3 and SCF-4 compounds. The inadequate blending of resin and NF in the polymer compounds may cause more spalling pits, debris and surface irregularity. However, SCF-1 and SCF-2 have shown higher contact plateaus, favorable for mixing all ingredients. KV-2 offers rougher surfaces with more wear debris and shear-induced texture than KV-1 composites. Furthermore, several fibers were found in SCF-3, while SCF-4 compounds pulled away from the polymer matrix, as shown in Figure 7. The chase test found that higher sugar fiber-based samples were easily worn when encountering hard grinding substances, which raised the wear rate [34,39,40,48]. However, KV-1 and SCF-1 display the smoothest surface of all compounds with small wear debris and spalling pits.

### 2.9. TGA of SCF-Based Experiments

**Figure 8:**
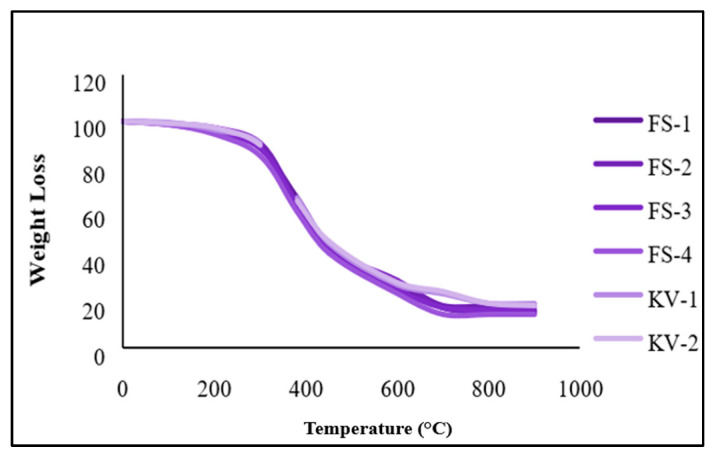
TGA of SCF and Kevlar-based compounds in the O_2_ environment.

The TGA has been performed with nitrogen and oxygen in two different environmental conditions. It has been noticed that the degradation occurred in three stages for both sugarcane and Kevlar composites. The weight reduction in the first stage has been seen as very low or less than 12%. In the second stage, a steep or heavy degradation has been noticed. At this stage, the losses were recorded by more than 77%. The third degradation shows a minimum loss in weight. The first, second and third degradation were reordered to 0–249 °C, 249–599 °C and 599–900 °C [41,42,43,44], respectively, as shown in Figure 8.

**Figure 9:**
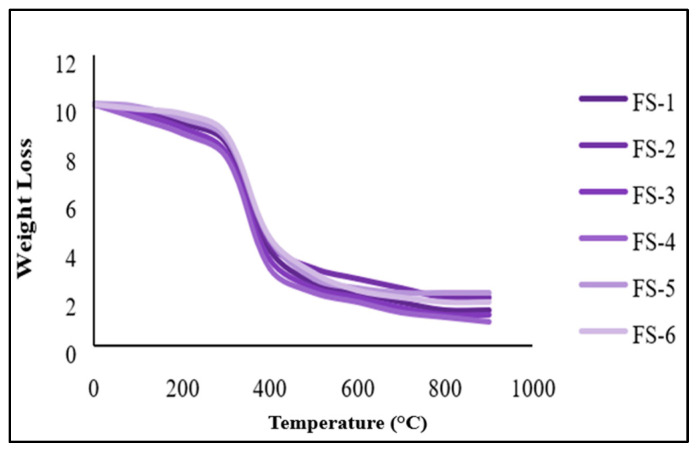
TGA of sugarcane and Kevlar-based composites at N_2_ atmosphere.

The degradation has been noted in a nitrogen environment from 0–299 °C, 299–599 °C and 600–900 °C, respectively, as shown in Figure 9 [42,43]. Heavy loss of more than 78% has been noted at 299–599 °C, where the first and third degradation shows minimum losses, less than 11%. The first degradation occurs due to the moister substance in the fiber and Kevlar. Secondly, degradation might be the reason for the decrease in hydrogen bonding in Kevlar compounds, the deficit of hemicelluloses and the discharge of gases from the SCF compounds. The third degradation occurs due to the amide group deficiency and loss of lignin and cellulose from KF and SCF [41,42,43,44,52,53,54,55,56]. The study has examined that raising the SCF and KF proportion in the compounds (5–20%) enhanced thermal stability [41,42,43,44]. The reach of the oxidation index under the O_2_ environment has been recorded between 5.40 and 5.63 and 5.70 and 5.84 in the N_2_ environment for SCF composites described in Table 3. In the case of Kevlar compounds, OI reach has been recorded between 5.60 and 5.65 in the O_2_ climate, while for the N_2_ environment, it was recorded at 5.80–5.85. However, for SCF-4, the thermal stability has been recorded as maximum, and for Kevlar-based composites, KV-2 displays more excellent thermal stability. The TGA of SCF and KF-based compounds in the N_2_ atmosphere are presented in Figure 9.

## 3. Materials and Methods

### 3.1. Compound or Composite Assembly

**Figure 10:**
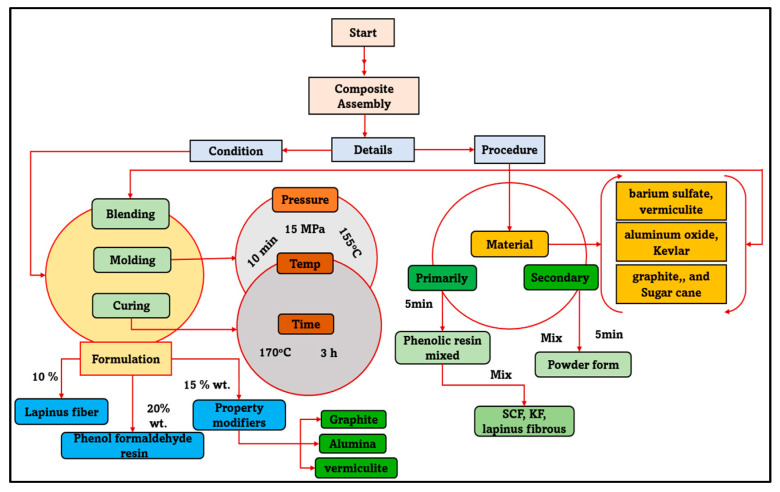
Composites assembly details.

The waste composition (fiber) has been accumulated from Himachal Pradesh; in India, fiber is cut out into 2–6 mm small sizes. Therefore, it has been treated with NaOH to remove the impurities from small pieces of thread [57,58]. The ingredients used in brake pads are barium sulfate, aluminum oxide, vermiculite, graphite and sugarcane. All these materials were appropriately mixed with the plow machine. After that, these materials were poured into the molding machine to develop brake pads [34,39]. The compositional detail, mixing and molding conditions are shown in Table 4 and Figure 10. Various materials were used in the composite formulation. The Novolac phenolic reis is manufactured using an acidic catalyst and a relative surplus of phenolic to formaldehyde. The simplified synthesis shows the enormous number of conceivable polymers. The first reaction takes place between methylene glycol and phenol. A standard phenol novolac resin has an average molecular weight (Mn) of 250 and 900. Barium sulfate is an inorganic substance having the chemical formula BaSO_4_. It is a white crystalline material that is notoriously insoluble in water. It may be found in mineral barite, the primary commercial barium source. Its significant applications take advantage of its opaque white appearance and high density. In the composite, barium sulfate D-1.0 (98%min) was used. Aluminum oxide (Al_2_O_3_) is a chemical compound of aluminum and oxygen. It is the most common of many aluminum oxides and is known as aluminum oxide. The aluminum oxide used in the composite has a molecular weight of 101.961. Kevlar fiber is intrinsically durable at higher temperatures, with slight shrinkage, little creep and a high glass transition temperature. It is corrosion-resistant, non-conductive and chemically resistant to all but solid acids and bases. The properties of Kevlar used in the composite have a tensile strength of 23 gpd, initial modulus of 550 gpd, elongation of 3.6%, a density of 1.44 g/cc and moisture regain of 6%.

### 3.2. Physico-Mechanical and Thermal Attributes

Mechanical assets like ultimate tensile strength (UTS), proof stress, ultimate compressive strength (UCS), total elongation and tensile modulus have been determined by a universal testing machine (UTM) observed by the ASTM E8 standard. The sample size has been taken with (70 × 20 × 10) mm dimensions. However, the impact energy test has been accomplished on the pendulum impact testing machine at GIET, Solan. UTM has been used to calculate the strength at three points and the modulus (flexure) of the samples as per the ASTM ED790 standard. Hind-type UTM (Allied Nippon, Ghaziabad, India) has been used to calculate the shear strength. The uncured resin has been evaluated with acetone extraction instruments [40]. The density has been determined with a sensor weighing scale [39]. The specimens were dipped in preheated oil (SAE 90) to discover the permeability of the prepared samples [39]. The manufactured pieces (2–4 g) were burnt in the furnace for 2 h at a temperature of 860 °C to get the ash content. As per ASTM D 570-98 standard to calculate the water absorption, the developed brake pads are plunged into distilled water for 24 h [41]. However, the pendulum impact testing machine has been used at GHEC, Solan, for the impact strength of the specimen calculation. The compressibility testing machine has been applied to achieve compressibility corresponding to ISO 6310 Standard [42]. A weighing device has been used to discover the alterations in the weight of the experiment sample, i.e., water absorption and ash contents test. However, the hardness calculates the R ratio on the harness tester (model: TRSN). Fuel instruments supply UTM, and engineering has been applied to examine the shear strength. To find heat swelling (%), samples were heated in a muffle furnace for 5 h at 50 °C temperature; tests were carried out as per 38SAE J 160 JNU80 standard, and sample dimension was taken (25 × 10 × 5) mm [42,43,44].

TGA was carried out on approximately 10 ± 0.1 mg of fabricated friction composite material on a TA-60WS model supplied by Shimadzu scientific instruments at a heating rate of 10 °C/min from 50 °C temperature to 800 °C in the presence of air and nitrogen atmosphere with the flow rate of 40 mL/min. Oxidation Index (OI) was calculated based on the weight of carbonaceous char (CR) [41,42,43,44]. The thermo-gravimetric analysis results are presented in Table 5.

### 3.3. Assessment of Tri-Biological Attributes of Established Compounds

A chase machine was used [34,39,40] for the experimental conditions shown in Table 6. The various tribological proprieties have been assessed through the analysis, i.e., friction fluctuation (Δμ), wear, stability coefficient (μ_s_), fade (%), performance coefficient (μ_p_), variability coefficient (μ_v_), recovery (%).

## 4. Conclusions

Non-asbestos sugarcane fiber-containing brake pads were fabricated to study the physical, chemical, thermal, mechanical and tri-biological properties. The result achieved from the study has been presented as follows. The sample’s water absorption, porosity and compressibility increased with a higher proportion of SCF in the compound. However, adding SCF proportion and hardness in developed mixtures decreases the ash proportion and hardness value.

(1) The tensile strength, ultimate compressive strength, flexural strength, tensile modulus, flexural modulus and impact energy revealed the most excellent performance for SCF-1 (5 wt.%) brake pad composite, including all SCF composites. The SCF brake pads have shown the most mechanical results near Kevlar-based compounds. It has also been observed that the percentage of wear and friction level increases with the addition of SCF in the mix.
(2) The ultimate shear strength and proof stress revealed the best performance for SCF-2 brake pad compounds, including the entire SCF composites. In contrast, Kevlar composites have shown their maximum value of 5 wt—% (KV-1) compounds. SCF-1 sample showed the highest and lowest values of µs and µ, respectively.
(3) The value of µs enhances with SCF proportion, while µ_v_ decreases with an increasing proportion of SCF. The SCF compounds (SCF-4) showed the ultimate value of recovery percentage. Although, the (%) recovery has been discovered to be over the hundred percent for all the desirable compounds. SCF-1 showed the utmost value of the performance of COF, with minor wear.
(4) The comparative study determined that the Kevlar-based brake pad samples revealed slightly improved %-fade, µ, wear and thermal stability results than SCF and KV-2 compounds. Thermal stability for SCF compounds exhibited the maximum value of OI (5.84) at 20 wt—% SCF composites. From the analysis, it has been concluded that SCF-based brake pad samples indicated excellent performance in physical and chemical mechanical properties and noticed values very near Kevlar composites. Moreover, SCF-1 compounds exhibit improved thermal, tri-biological and mechanical outcomes among all SCF compounds.
(5) The worn surface study reveals that, in addition to abrasive and adhesive wear processes, the development of primary and secondary contact patches/plateaus is one of the most critical elements in determining the tribological performance of the composite.

## Figures and Tables

**Table 2:** Attributes utilized in the tri-biological application.

Favorable Value of Elements							
µP	µv	%Fade	µs	% R	µR	µF	Wear rate
µ-standard	Least	Small	Larger	Larger	Larger	Small	Least

**Table 3:** Residue and oxidation index for SCF and Kevlar compounds.

Samples	SCF-4	SCF-3	SCF-2	SCF-1	KV-1	KV-2
% residue (O_2_) of SCF compounds	80.5	79.8	78.9	77.4	80.7	79.7
Oxidation index of SCF composites	5.63	5.58	5.55	5.40	5.65	5.60
% residue (N_2_) of SCF composites	83.70	83.45	82.69	82.58	83.72	82.9
Oxidation index (OI) N_2_	5.84	5.76	5.72	5.70	5.85	5.80

**Table 4:** Composition aspect of materials.

Sample	SCF-1	KV-1	SCF-2	KV-2	SCF-3	SCF-4
KF (wt.%)	-	05	-	10	-	-
BaSO_4_ (wt.%)	50	50	45	45	40	35
SCF (wt.%)	05	-	10	-	15	20
Composition	45	45	45	45	45	45

Formulation = Phenol formaldehyde resin (10%), lapinus fiber (20 wt.%), property modifiers (graphite, alumina and vermiculite = equal ratio (15 wt.%).

**Table 5:** Materials TGA assessment.

Instrument	Shimadzu Scientific Instruments
Fabricated friction compound on TA-60WS model	10 ± 0.1 mg (around)
Heating ratio	50 °C to 800 °C and 10 °C/min
Flow value	40 mL/min (air and nitrogen)
Calculation of oxidation index (OI)	Vary with a weight of carbonaceous char (CR)

**Table 6:** Experimental conditions of tribological proprieties (IS 2742-4 Standard).

Test Parameters	Application Applied	Heating (Status)	Applied Load (N)	Speed (rpm)	Max. Temp. (°C)	Time (min.)	Min. Temp. (°C)
Primary fade	1	off	440	308	93	20	-
Primary recovery	1	on	660	411	289	10	82
Secondary fade	1	off	660	411	261	10	93
Secondary recovery	1	off	660	411	317	10	82
Burnish	1	off	660	411	345	10	93

## Data Availability

The data used to support the findings of this study are included in the article.
